# Plant Cadmium Resistance 2 (SaPCR2) Facilitates Cadmium Efflux in the Roots of Hyperaccumulator *Sedum alfredii* Hance

**DOI:** 10.3389/fpls.2020.568887

**Published:** 2020-10-30

**Authors:** Jiayu Lin, Xiaoyu Gao, Jianqi Zhao, Jie Zhang, Shaoning Chen, Lingli Lu

**Affiliations:** ^1^Key Laboratory of Environment Remediation and Ecological Health, College of Environmental & Resource Science, Zhejiang University, Hangzhou, China; ^2^College of Life Sciences and Medicine, Zhejiang Sci-Tech University, Hangzhou, China; ^3^Key Laboratory of Subtropic Soil and Plant Nutrition, College of Environmental & Resource Science, Zhejiang University, Hangzhou, China

**Keywords:** cadmium, plant cadmium resistance family, hyperaccumulator, *Sedum alfredii*, efflux

## Abstract

Hyperaccumulators are the preferred materials for phytoremediation. *Sedum alfredii* Hance is a cadmium (Cd) hyperaccumulator plant in China, although its detoxification mechanism remains unresolved. In our study, we cloned a gene belonging to the plant cadmium resistance (*PCR*) family, named *SaPCR2*, from the hyperaccumulating ecotype (HE) of *S. alfredii*. Sequence analysis indicated that *SaPCR2* contained a cysteine-rich domain highly conserved in the *PCR* family and played an important role in Cd detoxification. Based on the relative quantitative results, *SaPCR2* was highly expressed in the roots of HE *S. alfredii*, but not the shoots and Cd exposure did not significantly affect *SaPCR2* expression. In contrast, the expression level of *SaPCR2* was very low in plants of its non-hyperaccumulating ecotype (NHE). The subcellular localization of SaPCR2 in tobacco leaves and yeasts showed that SaPCR2 was localized on the plasma membrane and the expression of the SaPCR2 protein in a Zn/Cd-sensitive yeast Δ*zrc1* significantly increased its tolerance to Cd stress by decreasing the Cd content in cells. Heterologous expression of *SaPCR2* in plants of both *Arabidopsis thaliana* and NHE *S. alfredii* significantly reduced the Cd levels in the roots, but not in the shoots. These results suggest that the overexpression of *SaPCR2* in plants provides a route for Cd leak out of the root cells and protects the root cells against phytotoxicity of Cd stress. To the best of our knowledge, this is the first study of transporter-mediated root efflux of Cd in hyperaccumulator *S. alfredii*.

## Introduction

Cadmium (Cd) is one of the most toxic elements for plants, animals, and human beings. High Cd exposure to crops via the biogeochemical behavior of soil-plant systems significantly threatens human health (Godt et al., 2006; Marisela Fernandez-Valverde et al., 2010; Shahid et al., 2017). During the past few decades, heavy metals (including Cd) have become the greatest pollutants worldwide due to the rapid development of the chemical industry, metallurgy, and electroplating (Hu et al., 2016). For instance, Cd is reported to be the most severe pollutant for agricultural soils in China (Li et al., 2014; Zhao et al., 2015; Shi et al., 2019). Phytoremediation of heavy metals is an efficient and economical soil remediation technique to clear or alleviate heavy metal pollution in soils (Liu et al., 2018). Hyperaccumulators can accumulate 100–1,000-fold higher heavy metals than normal plants without toxic consequences (Rascio and Navari-Izzo, 2011) and, therefore, are of high scientific interest as a representative plant material to extract heavy metals with great ability (Pollard et al., 2014).

To grow healthily in highly contaminated soils, a sophisticated detoxification system must exist in the plants of hyperaccumulators, especially for the root systems, which suffer first from heavy metal toxicity. Therefore, high root tolerance is the precondition for hyperaccumulating plants to grow well under high Cd stress. Roots of hyperaccumulators might present superior antioxidative defenses under Cd condition (Boominathan and Doran, 2003; Chiang et al., 2006; Wu et al., 2015) or sequestrated Cd in vacuoles and cell walls (Fu et al., 2011; Parrotta et al., 2015). However, the most important strategy for the root resistance of hyperaccumulators is to efflux Cd out of the root cells and subsequently translocate to the aboveground parts of plants. In hyperaccumulator *Noccaea caerulescens*, the great ability of xylem loading is one of the main processes participating in Cd hyperaccumulation (Sterckeman et al., 2015). High expression of *HMA4* in *Arabidopsis halleri* supports the enhanced metal flux from the root symplasm into the xylem vessels necessary for shoot metal hyperaccumulation (Hanikenne et al., 2008). This is different from the strategies of some regular or resistant plants to prevent heavy metals from accumulating in plants. For example, OsZIP1 can function as a metal exporter in rice when zinc (Zn), copper (Cu), and Cd are present in excess in the environment and decrease the metal accumulation in plants (Liu et al., 2019). Therefore, the investigation of transporters involved in the efflux of Cd in the roots of hyperaccumulators may facilitate our understanding of metal tolerance in roots as well as its hyperaccumulation in shoots.

Plant cadmium resistance (PCR) family transporters, especially *PCR1* and *PCR2*, are involved in the efflux of Cd and other bivalent cations from cells to the outside, mainly via the lateral transport and xylem loading in the roots. The first PCR family gene found in *A. thaliana*, named *AtPCR1*, showed a strong tolerance for Cd in the selection of Cd-tolerant genes of *A. thaliana* using yeast Cd-sensitive mutants (Song et al., 2004). The study of OsPCR1 in rice showed that the *OsPCR1* knockdown lines decreased the weight and Cd content of grains (Song et al., 2015). AtPCR2 is mainly distributed on the epidermis of the root hair zone, as well as the vascular and epidermal cells of the elongation zone, which plays a very important role in the translocation of Zn (Song et al., 2010). In *Brassica juncea*, BjPCR1 was found to be localized on the plasma membrane of the root epidermal cells at the subcellular level and is mainly responsible for the absorption and root-to-shoot transportation of calcium (Ca) (Song et al., 2011). However, to the best of our knowledge, no study has been undertaken on the function of the PCR family protein in hyperaccumulating plants.

*Sedum alfredii* Hance, a Chinese native Zn/Cd hyperaccumulator belonging to the Crassulaceae family (Yang et al., 2004, 2006), has a high capacity for tolerating, translocating, and accumulating high amounts of Cd (Lu et al., 2008; Tian et al., 2011, 2017). The Cd concentration in the shoots of HE *S. alfredii* is as high as 9,000 mg/kg (Yang et al., 2004). However, the molecular mechanisms involved in root resistance and translocation of Cd remain unknown. In the present study, a high expression gene belonging to the PCR family, named *SaPCR2*, was cloned from this plant species and the functions of this gene were investigated.

## Materials and Methods

### Plant Materials and Growth Conditions

The hyperaccumulating ecotype (HE) *S. alfredii* and its non-hyperaccumulating ecotype (NHE) were originally collected from a Zn/Cd mine in Quzhou (Zhejiang province, China) and tea plantation in Hangzhou (Zhejiang province, China), respectively. The plant materials used in this experiment were cultured after several generations of asexual cuttings. The same seedlings were cut and exposed to deionized water until rooting and were then added to 1/4, 1/2, and full nutrient solution gradually, which was replaced every 3 days. The formulation of the full nutrient solution contained 2.0 mM Ca(NO_3_)_2_, 0.7 mM K_2_SO_4_, 0.1 mM KH_2_PO_4_, 0.1 mM KCl, 0.5 mM MgSO_4_, 20 μM Fe-EDTA, 5 μM ZnSO_4_, 10 μM H_3_BO_3_, 5 μM ZnSO_4_, 0.5 μM MnSO_4_, 0.2 μM CuSO_4_, and 0.01 μM (NH_4_)_6_Mo_7_O_24_. The temperature of incubation was set at 25–28°C, with a 16 h light/8 h dark cycle. Three-week-old seedlings were used in subsequent experiments.

### *SaPCR2* Cloning and Sequence Analysis

The results of the previous transcriptome sequence of HE *S. alfredii* illustrated that the sequence (Sa_Contig10958, GenBank: HE728063) was predicted to encode the gene from the *PCR* family (Gao et al., 2013). According to this sequence (Sa_Contig10958), we designed RACE primers to clone the full-length sequence from HE and NHE *S. alfredii* using the Smart RACE cDNA amplification kit (Clontech). The cDNA sequences of *SaPCR2* are the same in two ecotypes (Supplementary Figure S1), so we named this gene as *SaPCR2*. The primer used for *SaPCR2* 5′-RACE was 5′-CACGGAGACTCTTGTAGATCATAC-3′ and the primer used for *SaPCR2* 3′-RACE was 5′-TGCCATCTACGGCCTGATTT-3′. The transmembrane domain of SaPCR2 was predicted using the TMHMM Online analysis tool^1^. The protein sequence alignment analysis was compared with ClustalX. The phylogenetic tree analysis was performed using MEGA5.0 with the neighbor-joining method.

### Expression Pattern of *SaPCR2*

Two ecological types of *S. alfredii* were treated by CdCl_2_ with different concentrations (HE: 10 and 100 μM, NHE: 10 μM) and times (0, 6, 24 h, 3, and 7 days). The roots, stems, and leaves of each plant were separated and frozen rapidly in liquid nitrogen. The total RNA was extracted using a Spin Column Plant Total RNA Purification Kit (Sangon) and then synthesized to cDNA with a HiScript II Q RT SuperMix for qPCR (+ gDNA wiper) (Vazyme). Real-time quantitative PCR (RT-qPCR) was performed using a ChamQ SYBR Color qPCR Master Mix (Without ROX) (Vazyme), with LightCycler 480 System (Roche, United States). The RT-qPCR protocol was as follows: 95°C for 3 min, 40 cycles of 95°C for 10 s, 60°C for 30 s. The melting-curve analysis was included to verify the specificity of the primer. The mean amplification efficiency was analyzed with the LinReg software (Ruijter et al., 2009). The specific primers for RT-qPCR were designed according to the *SaPCR2* sequence as follows: *SaPCR2* forward 5′-GCGGTGGGATGTGGTCTAC-3′ and *SaPCR2* reverse 5′-CGATAATCTCGGCTATTTGGC-3′, *SaACTIN1* forward 5′-TGTGCTTTCCCTCTATGCC-3′, and reverse: 5′-CGCTCAGCAGTGGTTGTG-3′ (Chao et al., 2010). The relative expression levels were calculated using 2^−Δ*Ct*^ method.

### Plasmid Construction

All the *SaPCR2* sequences for reconstruction were amplified using the ClonExpress II One Step Cloning Kit (Vazyme), and the primers used are listed in Table 1. To generate yeast expression vectors, the open reading frame of *SaPCR2* was cloned into the *Spe*I and *Eco*RI sites of the pDR196 (*pDR196-SaPCR2* vector), which contained a uracil amino acid selection marker. To produce the green fluorescent protein (*GFP*)-fused *SaPCR2* expression vector (*35S_*pro*_-SaPCR2-GFP* vector) for transient expression in tobacco, the open reading frame of *SaPCR2* sequence without stop codon was cloned into the pCAMBIA 1300-eGFP vector between the *Kpn*I and *Bam*HI sites, which was controlled by the cauliflower mosaic virus (CaMV) 35S promoter. Taking pCAMBIA 1300 as the plant overexpression vector, we cloned the full-length *SaPCR2* into the *Acc*I and *Xba*I restriction sites.

**TABLE 1:** Primers used for vector construction of *SaPCR2.*

Yeast expression vector (*pDR196-SaPCR2* vector)	Forward	tataccccagcctcgactagt ATGTATCCATCTTTGTCTG
	Reverse	gataagcttgatatcgaattc TCATCTACTCATCCCCTGC
GFP expression vector (*35S_*pro*_-SaPCR2-GFP* vector)	Forward	tacgaattcgagctcggtaccATGTATCC ATCTTTGTCTGAAAATGAG
	Reverse	catgtcgactctagaggatcc TCTACTCATCCCCTGCGGAA
Plant expression vector (*35S_*pro*_-SaPCR2* vector)	Forward	aagcttatcgataccgtcgac ATGTATCCATCTTTGTCTG
	Reverse	gggggatccactagttctaga TCATCTACTCATCCCCTGC

*The lowercase indicates recombinant sequences.*

### Subcellular Localization of SaPCR2

The tobacco leaves were used to observe the subcellular localization of SaPCR2. *Agrobacterium tumefaciens* GV3101 monoclonal containing the *35S_*pro*_-SaPCR2-GFP* vector was picked, activated, and suspended in 10 mM MgCl_2_ and 10 mM MES (pH = 5.6). The final OD_600_ of the bacteria concentration was adjusted to approximately 0.4 and it was then injected into the epidermis of the tobacco leaves using a needleless syringe, cultured for 24–36 h. The epidermis was treated with 0.8 M mannitol for at least 10 min to induce plasmolysis and the fluorescence was detected using a confocal microscopy (LSM700, Carl Zeiss, Germany). FM 4–64 fluorescence was used as a plasma membrane marker.

### Cd Resistance in Yeast Strains Expressing *SaPCR2*

To express the SaPCR2 in yeast, the *pDR196-SaPCR2* vector or pDR196 empty vector were transformed into the wild type BY4743 and Zn/Cd-sensitive mutant Δ*zrc1* (MAT1; his3; leu2; met15; ura3; YMR243c: kanMX4) using the LiAc/PEG/ssDNA method (Gietz and Schiestl, 2007). The positive strains were screened out by selected synthetic dropout (SD) solid media (absence of uracil amino acid) and verified by PCR with specific primers: forward 5′-GCGGTGGGATGTGGTCTAC-3′ and reverse 5′-CGATAATCTCGGCTATTTGGC-3′. For Cd tolerance assay, yeast cells were cultured in selected liquid SD until OD_600_ = 1. Then, 5 μL serial dilutions (OD_600_ = 1.0, 0.1, 0.01, and 0.001) were spotted on SD with 0, 10, and 60 μM CdCl_2_ at 30°C. Photographs were taken after 3-days incubation. For Cd accumulation testing, the yeast was cultured overnight to OD_600_ = 0.1 and then added to the SD liquid medium with 2.5, 5.0, and 10.0 μM CdCl_2_ for 24 h at 30°C. The yeast cells were collected via centrifugation and washed three times with ultra-pure water. The cells were dried for 2 days at 85°C and digested in HNO_3_ to determine the heavy metal concentration in the yeast strains by inductively coupled plasma mass spectrometry (Agilent, United States).

### Plant Transformations

To investigate *SaPCR2* in *S. alfredii*, the transgenic system of NHE *S. alfredii* was constructed. The seeds of NHE *S. alfredii* were sterilized in 0.1% HgCl_2_ for 5 min and washed with sterile water five to six times and were then spotted on 1/2 Murashige and Skoog (MS) medium (Murashige and Skoog, 1962). After approximately 8 weeks, young stems of the aseptic seedlings were cut into approximately 1–2 cm and transferred to callus-inducing medium (Table 2) with 6-benzylaminopurine (6-BA) and 1.0 mg/L 2,4-dichlorophenoxyacetic acid (2,4-D). When the stem segments appeared to be differentiated and enlarged, *A. tumefaciens* GV3101 (OD_600_ = 0.6) was used for transfection. Then the explants were co-cultured with *Ag. tumefaciens* containing the *35S_*pro*_-SaPCR2* vector for 2 days and then removed to the selection medium (Table 2). The hyg-resistant explants were moved to differential medium (Table 2). After emergence, the medium was changed to 1/2 MS-agar medium. Then, the well-rooted plants were moved to nutrient solution for subsequent experiments. *SaPCR2* overexpressing *A. thaliana* was obtained using the *Agrobacterium*-mediated floral dip method (Clough and Bent, 1998).

**TABLE 2:** Optimization of the transgenic system of *S. alfredii.*

Process	Medium	Main operation
Seed germination	1/2 MS	Sterilize seed and spot on medium for 8 weeks.
Induction	MS + 0.5 mg/L 6-BA + 1.0 mg/L 2,4-D	Cut stem into 1–2 cm sections and culture for 3 days.
Cocultivation	MS + 0.5 mg/L 6-BA + 1.0 mg/L 2,4-D + 100 mg/L acetosyringone (AS)	Cultivate bacterial solution until OD_600_ reaching 0.6, then resuspend by resuspension (MS + 100 mg/L AS) and infect for 15 mins. The total cocultivation time should not exceed 2 days.
Selection	MS + 0.5 mg/L 6-BA + 1.0 mg/L 2,4-D + 100 mg/L Timentin + 30 mg/L hygromycin (Hyg)	Soak the explants into three 100 mg/L Timentin for 15 mins, then wash three to four times using sterile water.
Differentiation	MS + 0.5 mg/L 6-BA + 0.1 mg/L 1-Naphthaleneacetic acid (NAA) + 100 mg/L Timentin + 30 mg/L Hyg	Cut healthy callus and transfer to differentiation medium, change medium once at 14 days.
Rooting	1/2 MS + 100 mg/L Tim + 30 mg/L Hyg	Move robust differentiated shoots to rooting medium.

The total plant genomic DNA was extracted from the fresh leaves of transgenic plants and their wild type using the CTAB method, identified by the following primers: forward 5′-ATGTATCCATCTTTGTCTG-3′ and reverse 5′- TCATCTACTCATCCCCTGC-3′. The expression level of *SaPCR2* in the transgenic NHE *S. alfredii* was investigated by RT-qPCR as mentioned above. Two independent transgenic lines of NHE *S. alfredii* (SaPCR2-L1, SaPCR2-L2) and *A. thaliana* of T2 plants (SaPCR2-OX1, SaPCR2-OX2) were identified as *SaPCR2* overexpressing lines for subsequent analysis.

### Cd Concentration of Plants

The seeds of the transgenic *A. thaliana* were collected and spotted on 1/2 MS solid medium and then 4-days-old seedlings were transferred to nutrient agar plates with 15 or 30 μM CdCl_2_ for 7 days. There were 20 plants for one treatment and each treatment was repeated three times. Well-rooted transgenic NHE *S. alfredii* lines and their corresponding wild type plants in the same growth states were hydroponically cultured for 3 weeks and then treated with 10 μM CdCl_2_ for 5 days. For Cd concentration determination, all the plant samples were rinsed in 20 mM Na_2_-EDTA to remove excess Cd^2+^ attached to the surface and were then separated into roots, stems, and leaves for drying and digesting in HNO_3_-H_2_O_2_. The Cd concentration was analyzed using an inductively coupled plasma optical emission spectrometer (Agilent, United States).

The Cd uptake (μg/kg root FW) = Total Cd in the plants (μg)/Fresh weight of roots (kg).

### Plasma Membrane Integrity and Lipid Peroxidation of Roots

The plants were exposed to 10 μM CdCl_2_ for 7 days. The plasma membrane integrity and lipid peroxidation of roots were investigated using the analysis of Evans blue uptake, MDA content, as well as Schiff’s reagent staining (Tian et al., 2012).

## Results and Discussion

### Sequence Analysis of SaPCR2

**FIGURE 1:**
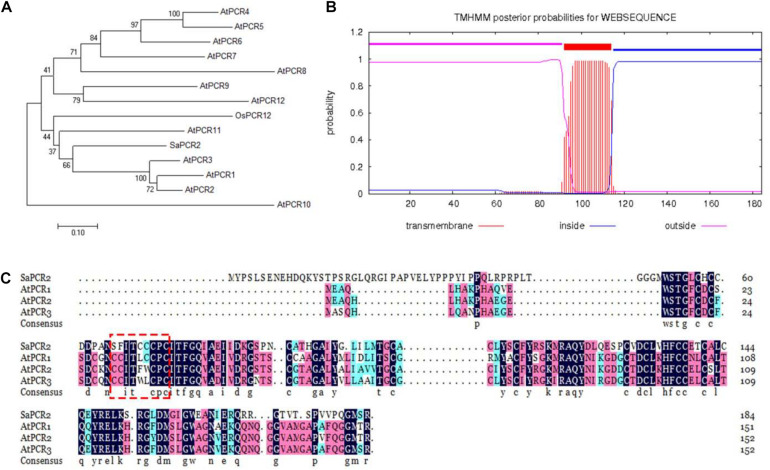
Sequence analysis of SaPCR2. **(A)** Phylogenic tree of SaPCR2 proteins in *Arabidopsis thaliana*, *Oryza sativa*, and *Sedum alfredii*. The 0.1 scale showed substitution distance. **(B)** SaPCR2 protein transmembrane topology prediction. **(C)** Amino acid sequence alignment of SaPCR2, AtPCR1, AtPCR2, and AtPCR3. Identical residues are shown in black, residues with a similarity over 70% are shown in red, and 50% or more are shown in blue. The fully conserved CCXXXXCPC domains are framed by the red box.

As an unessential element, Cd is suggested to enter/exit plant cells through the transporters of bivalent cations for nutrient elements, such as Zn/Mn/Fe (Ueno et al., 2008; Willems et al., 2010; Sasaki et al., 2012). The PCR family has been confirmed as membrane transporters not only for the transportation of Cd, but also for Zn, Fe, and Ca (Song et al., 2004, 2010, 2011). In this study, the putative *PCR* gene was cloned from *S. alfredii* according to the published transcription sequence (Gao et al., 2013). Phylogenetic analysis revealed that the putative PCR amino acid sequence was 44.68% similar to AtPCR2 belonging to *A. thaliana* (Figure 1A). Therefore, we named this PCR transporter *SaPCR2*. The SaPCR2 protein sequence contained 184 amino acids and a transmembrane domain predicted with the TMHMM Online analysis tool (Figure 1B). A multiple sequence alignment with PCR from *A. thaliana* was undertaken, showing that the SaPCR2 protein exhibited the same familial signature (Figure 1C). Analysis of the amino acid sequences showed that SaPCR2 contained a highly conserved CCXXXXCPC domain. This small cysteine-rich protein may play an important role in the detoxification mechanisms of Cd (Song et al., 2004). Therefore, SaPCR2 identified in the HE *S. alfredii* may be of great importance in Cd transportation.

### *SaPCR2* Is Highly Expressed in the Roots of HE *S. alfredii*

**FIGURE 2:**
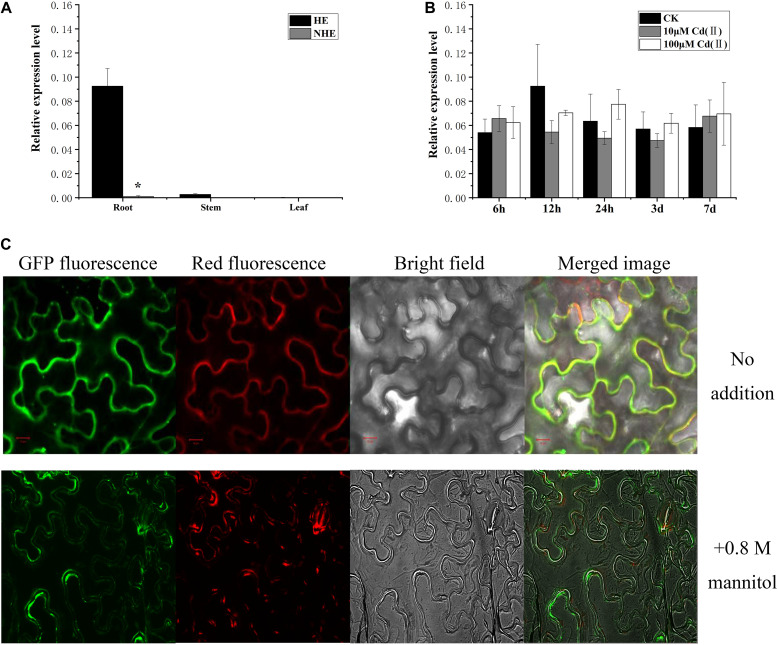
Expression of *SaPCR2* and subcellular location of SaPCR2. **(A)** Expression level of *SaPCR2* in roots, stems, and leaves of two ecotypes (HE and NHE) of *Sedum alfredii*. **(B)** Expression levels of *SaPCR2* in the roots of the HE *S. alfredii* at different time points. The plants were treated with 0, 10, or 100 μM CdCl_2_ for 6, 12, 24 h, 3, or 7 days. Error bars are ± SE of *n* = 3. Relative expression levels were normalized to the level of constitutively expressed gene *SaACTIN1*. ^∗^Indicates significant difference between ecotypes (*p* < 0.05). **(C)** Subcellular location of SaPCR2 in epidermal cells of tobacco. Distribution of green fluorescent protein (GFP)-SaPCR2 fusion protein and red fluorescent protein (plasma membrane marker) in tobacco leaf epidermis cells. Scale bar = 10 μm.

To understand the expression characteristics of *SaPCR2* gene, we analyzed its expression in the roots and shoots of the two *S. alfredii* ecotypes using RT-qPCR. High expression of *SaPCR2* was observed in the roots of the HE *S. alfredii*, but not in the shoots (Figure 2A). The expression level of *SaPCR2* in the roots was approximately 33-fold higher than that in the stems of the HE (Figure 2A). This differed from that of *AtPCR1*, which was exclusively expressed in the aboveground parts of *A. thaliana* (Song et al., 2004), yet similar to that of *BjPCR1* in *Brassica juncea* (Song et al., 2011). To investigate the effects of Cd exposure on *SaPCR2* expression, we analyzed the variations in the *SaPCR2* expression levels in the HE roots after different Cd exposure for 6 h to 7 days. Cd exposure did not result in any significant variation of the *SaPCR2* expression levels in the HE roots (Figure 2B). Therefore, this gene is not induced by Cd stress. In plants of NHE *S. alfredii*, the *SaPCR2* expression was not observed in either roots or shoots regardless of Cd treatments (Figure 2A).

These results are similar to the transcript levels of several reported Cd-related transporters in plants of HE *S. alfredii*. The high expression of these genes may result from the adaption of high Cd exposure during long-term evolution. For example, *SaHMA3* plays an essential role in Cd detoxification and its expression in HE *S. alfredii* was also significantly higher than that in NHE plants (Zhang et al., 2016), however, no significant difference was observed after Cd treatment (Liu et al., 2017). The high expression levels of these transporters in plants of HE *S. alfredii* may result from the long-term adaption and evolution of this plant species exposed to the high heavy metal levels in its natural habitat soils.

### SaPCR2 Localizes to the Plasma Membrane and Enhances Cd Resistance in Yeast

To determine the subcellular location of SaPCR2, *GFP* fused to *SaPCR2* under the control of the CaMV 35S promoter (*35S_*pro*_-SaPCR2-GFP*) construct was generated for the transient expression in epidermal cells of tobacco leaves. The green fluorescence signal of the SaPCR2-GFP fusion protein was observed in the plasma membrane showing co-localization with the plasma membrane marker dye FM 4-64 (Figure 2C). When exposed to a high concentration of sucrose, the green fluorescence signal was in close to the cell wall, but not organelle membrane, which was similar with BjPCR1 (Song et al., 2011).

**FIGURE 3:**
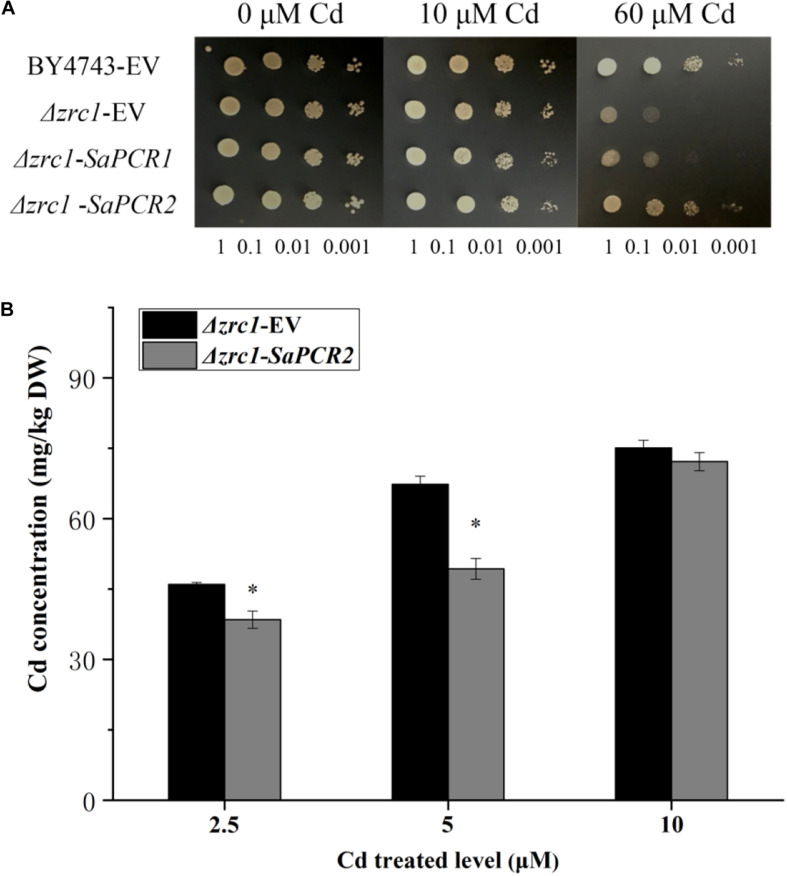
Expressing *SaPCR2* enhanced cadmium (Cd) tolerance in yeast mutant Δ*zrc1* by reducing Cd concentration. **(A)** The yeast strain BY4743 and Δ*zrc1* transformed with *pDR196-SaPCR2* vector or *pDR196* empty vector (EV) were diluted (OD_600_ = 1.0, 0.1, 0.01, and 0.001) and spotted on SD medium with 0, 10, and 60 μM CdCl_2_ for 3 days at 30°C. **(B)** Cd concentration in yeasts. Δ*zrc1* strains expressed EV and *pDR196-SaPCR2* were cultured in SD liquid medium with 2.5, 5, and 10 μM CdCl_2_ for 24 h at 30°C. Error bars are ± SE of *n* = 4. ^∗^Indicates significant differences between Δ*zrc1*-EV and Δ*zrc1-SaPCR2* (*p* < 0.05).

The effect of SaPCR2 on Cd resistance was assayed in Δ*zrc1* yeast strains. The *SaPCR2* transcript was recombined in the yeast expression vector pDR196. In the absence of Cd, there was no significant difference between the transgenic yeast strains (Figure 3A). On the SD solid medium supplemented with 60 μM CdCl_2_, the growth of the empty vector (EV) and *SaPCR2*-expressing lines in the Δ*zrc1* backgrounds were inhibited. However, the Δ*zrc1* strain transfected with EV showed higher sensitivity to Cd than the Δ*zrc1* strain expressing *SaPCR2* (Figure 3A). To test the Cd-transporting activity of SaPCR2, we determined the Cd concentration in the *SaPCR2*-expressing Δ*zrc1* mutant strain treated with Cd. When cultured in the presence of 2.5 or 5.0 μM CdCl_2_, SaPCR2-expressing Δ*zrc1* cells accumulated lower Cd than EV-transformed control cells (*p* < 0.05) (Figure 3B). These results indicate that SaPCR2 enhanced yeast Cd resistance by reducing Cd concentration in cells. Therefore, the SaPCR2 may function as a transporter at plasma membrance in the roots of HE *S. alfredii*, which mediates Cd reduction in cells.

### Heterologous of *SaPCR2* Reduced Cd Accumulation in *A. thaliana* and NHE *S. alfredii*

**FIGURE 4:**
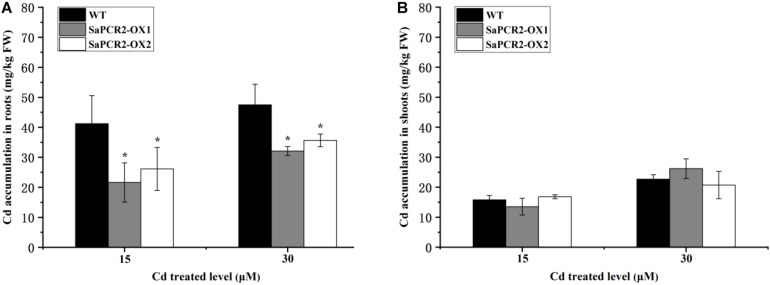
Cd concentration in *SaPCR2* overexpressing *Arabidopsis thaliana*. Cd concentration of **(A)** roots and **(B)** shoots in wild type, OX1, and OX2. Plants were cultured on 1/2 MS solid medium with 15 and 30 μM CdCl_2_ for 7 days, every 20 plants for one treatment, each treatment repeated three times. Error bars are ± SD of *n* = 3. *Indicates significant difference between genotypes (*p* < 0.05).

To further determine the effects of *SaPCR2* under Cd stress, we overexpressed *SaPCR2* in *A. thaliana*. Two lines transfected with *SaPCR2* (OX1 and OX2) were selected for subsequent experiments (Supplementary Figure S2A). The root lengths of transgenic lines were similar to the wild type in each treatment (Supplementary Figures S2B,C). However, the overexpressed *SaPCR2* significantly decreased Cd concentration in the roots compared with the wild type when plants were grown with 15 or 30 μM CdCl_2_ (*p* < 0.05) (Figure 4). The Cd accumulations in the roots of two independent overexpressing lines were 27–37% and 18–21% lower than the wild type, respectively (Figure 4A). By contrast, there was no significant difference in the accumulations of Cd in the shoots (Figure 4B). These results suggest that the overexpressed *SaPCR2* decreased Cd accumulation in the roots of *A. thaliana*.

**FIGURE 5:**
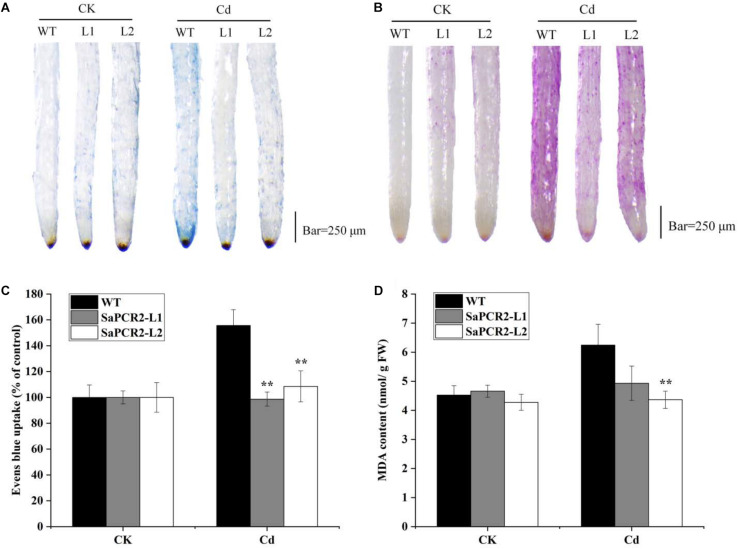
Cd-induced loss of plasma membrane integrity **(A,C)** and lipid peroxidation **(B,D)** in the root tips of *SaPCR2* overexpressing NHE *Sedum alfredii* line (L1 and L2). The plants were cultured in nutrient solution with 10 μM CdCl_2_ for 7 days, each treatment was repeated three times. Error bars are ± SD of *n* = 3. **Indicates significant difference between genotypes (*p* < 0.01). Scale bar = 250 μm.

**FIGURE 6:**
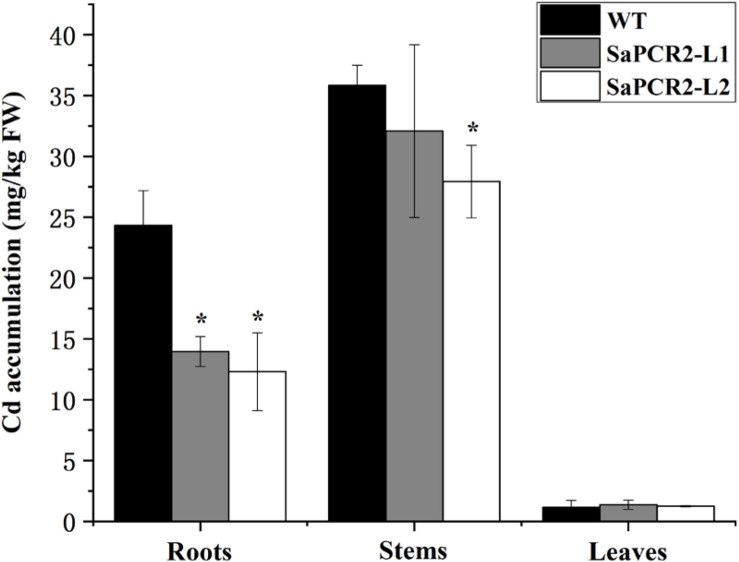
Cd concentration in the root, stem, and leaf of wild type and *SaPCR2*-overexpressed transgenic NHE *Sedum alfredii* lines (L1 and L2). The plants were cultured in nutrient solution with 10 μM CdCl_2_ for 5 days, each treatment was repeated three times. Error bars are ± SD of *n* = 3. *Indicates significant difference between genotypes (*p* < 0.05).

Due to the much higher expression levels of *SaPCR2* in HE plants than that of NHE, we generated *SaPCR2* NHE *S. alfredii* overexpressing lines (L1 and L2) to test the effect of *SaPCR2* in NHE plants under Cd exposure. Higher expression levels of *SaPCR2* were observed in L1 and L2. Compared with the wild type, the transcript levels of the *SaPCR2* in the roots of overexpressing lines were 109–114-fold higher (Supplementary Figures S3A,B). After culturing in nutrient solution with 10 μM CdCl_2_, the leaves of wild type (NHE *S. alfredii*) wilted and the biomass showed a downtrend (Supplementary Figure S3D), whereas the L1 and L2 plants showed little toxicity phenomenon, especially in the roots (Figure 5). The Cd concentrations in the roots of the wild type were significantly higher than those of the *SaPCR2* overexpressing lines (*p* < 0.05) (Figure 6). In the stems, L2 contained approximately 26% lower concentrations than the wild type; however, there was no significant difference in the leaves (Figure 5). Therefore, the heterologous overexpressing of *SaPCR2* significantly reduced Cd accumulation in the roots of the transgenic NHE *S. alfredii*, without translocating increased Cd to its shoots.

### Overexpression of *SaPCR2* Provides a Route to Leak Cd Out of the Root Cells

**FIGURE 7:**
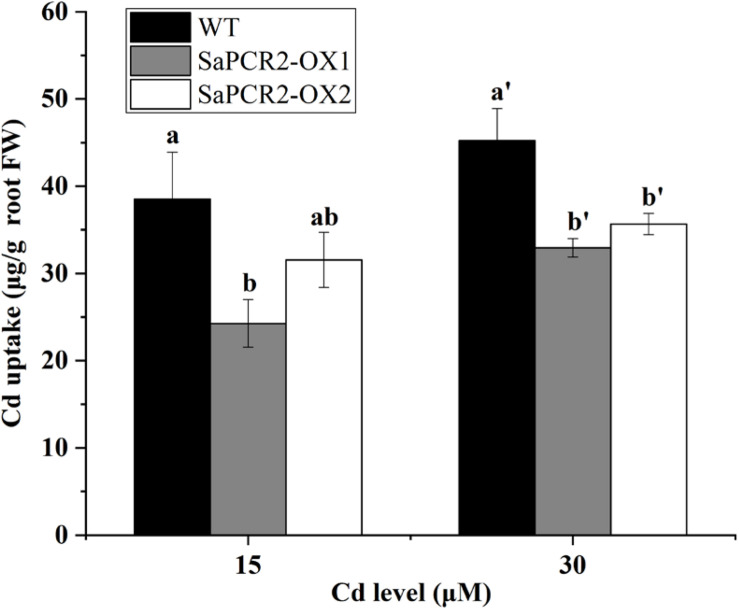
Cd uptake in wild type and *SaPCR2*-overexpressed transgenic *Arabidopsis thaliana* lines (OX1 and OX2). The plants were cultured on 1/2 MS solid medium with 15 and 30 μM CdCl_2_ for 7 days, every 20 plants for one treatment, each treatment repeated three times. Error bars are ± SE of *n* = 3. Different letters indicate significant differences between genotypes (*p* < 0.05).

**FIGURE 8:**
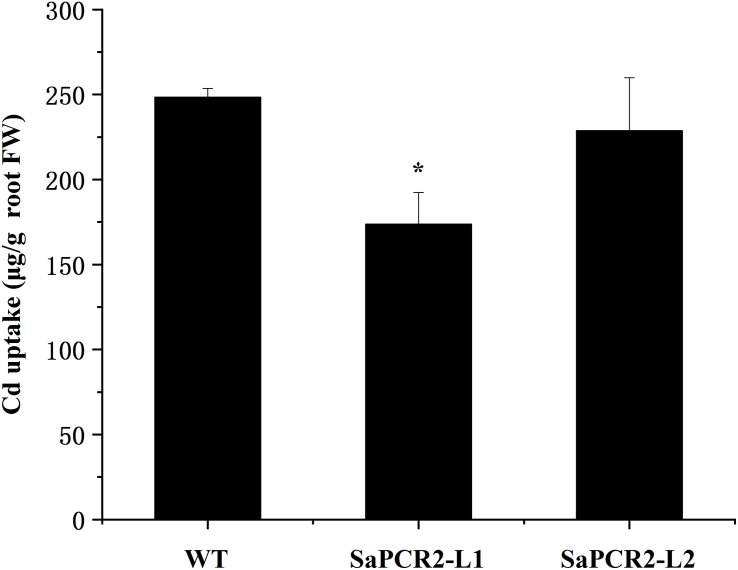
Cd uptake in wild type and *SaPCR2*-overexpressed transgenic NHE *Sedum alfredii* lines (L1 and L2). The plants were cultured in nutrient solution with 10 μM CdCl_2_ for 5 days, each treatment repeated three times. Error bars are ± SE of *n* = 3. ^∗^Indicates significant difference between genotypes (*p* < 0.05).

Currently, many studies on the physiological mechanisms of Cd tolerance in the roots of hyperaccumulators have been undertaken, which have shown three main mechanisms in the roots of HE *S. alfredii* that play an important role in Cd homeostasis, including (1) transportation: Cd could be transformed from root to shoot to avoid excess Cd accumulation in roots (Lu et al., 2008, 2009; Tian et al., 2017); (2) segmentation: HE *S. alfredii* can transfer Cd from the cytoplasm to the vacuole or cell wall (Tian et al., 2010, 2017); (3) chelation and anti-oxidation: small molecule compounds and intracellular Cd^2+^ are combined to reduce the ion concentration and the antioxidant system is activated to alleviate the Cd stress at the same time (Tian et al., 2012). Many transporters have been used to testify the mechanisms mentioned above. For example, ZIP, HMA, or YSL proteins are implicated in Cd transport across membranes (Gallego et al., 2012). P-type ATPases HMA2 and HMA4, localizing to the plasma membrane, play essential roles in controlling Cd translocation (Hussain et al., 2004; Wong and Cobbett, 2009; Nocito et al., 2011). CAL1 acts by chelating Cd in the cytosol and facilitating Cd secretion to extracellular spaces, thus lowering the cytosolic Cd concentration while driving long-distance Cd transport via xylem vessels (Luo et al., 2018). Therefore, in hyperaccumulating plants, the capacity to translocate Cd to the shoots is a comprehensively proved mechanism involved in the tolerance of the roots. However, further calculation showed that overexpressing *A. thaliana* and NHE *S. alfredii* lines of *SaPCR2* had lower Cd uptake than the wild type (Figures 7, 8). Therefore, the Cd decrease in roots not only depends on the efficient translocation systems, but is also attributed to the limit of Cd uptake and accumulation. For plants, there are two approaches to reduce the Cd concentration in roots—rapid root-to-shoot translocation and efficient efflux out of plants, both of which essentially protect the roots from too much Cd poison. For instance, heavy metal P-type ATPase OsHMA6 in rice, which is likely a Cu efflux protein (Zou et al., 2020). Therefore, we proposed a mechanism that has not been reported in the mechanism of Cd tolerance in HE *S. alfredii*, “leaking Cd out of plants.”

## Conclusion

The present study identified a gene coding SaPCR2 from HE *S. alfredii* expressed in roots. This transporter was localized to the plasma membrane. Heterologous overexpression of *SaPCR2* reduced Cd uptake and accumulation in plants. Therefore, these findings improve the Cd detoxification mechanisms of hyperaccumulators and may contribute to the development of phytoremediation and food safety in the future.

## Data Availability Statement

The datasets presented in this study can be found in online repositories. The names of the repository/repositories and accession number(s) can be found in the article/Supplementary Material.

## Author Contributions

XG, JL, JiaZ, and JieZ performed the experiments. JL, LL, and SC wrote and revised the manuscript. LL designed, supervised, and obtained funding for the project. All authors gave final approval for publication.

## Conflict of Interest

The authors declare that the research was conducted in the absence of any commercial or financial relationships that could be construed as a potential conflict of interest.

## Footnotes

1 http://www.cbs.dtu.dk/services/TMHMM/

## Abbreviations

HE: hyperaccumulating ecotype
NHE: non-hyperaccumulating ecotype
PCR: Plant cadmium resistance.
