# Supplementary material for: Plant Cadmium Resistance 2 (SaPCR2) Facilitates Cadmium Efflux in the Roots of Hyperaccumulator Sedum alfredii Hance
Source: Front Plant Sci. 2020 Oct 30;11:568887. doi: 10.3389/fpls.2020.568887 (PMC7661388; doi:10.3389/fpls.2020.568887)
Supplement: Supplementary file 1 [file Data_Sheet_1.docx]

**Figure S1.** The comparison of SaPCR2 cloned from HE and NHE.


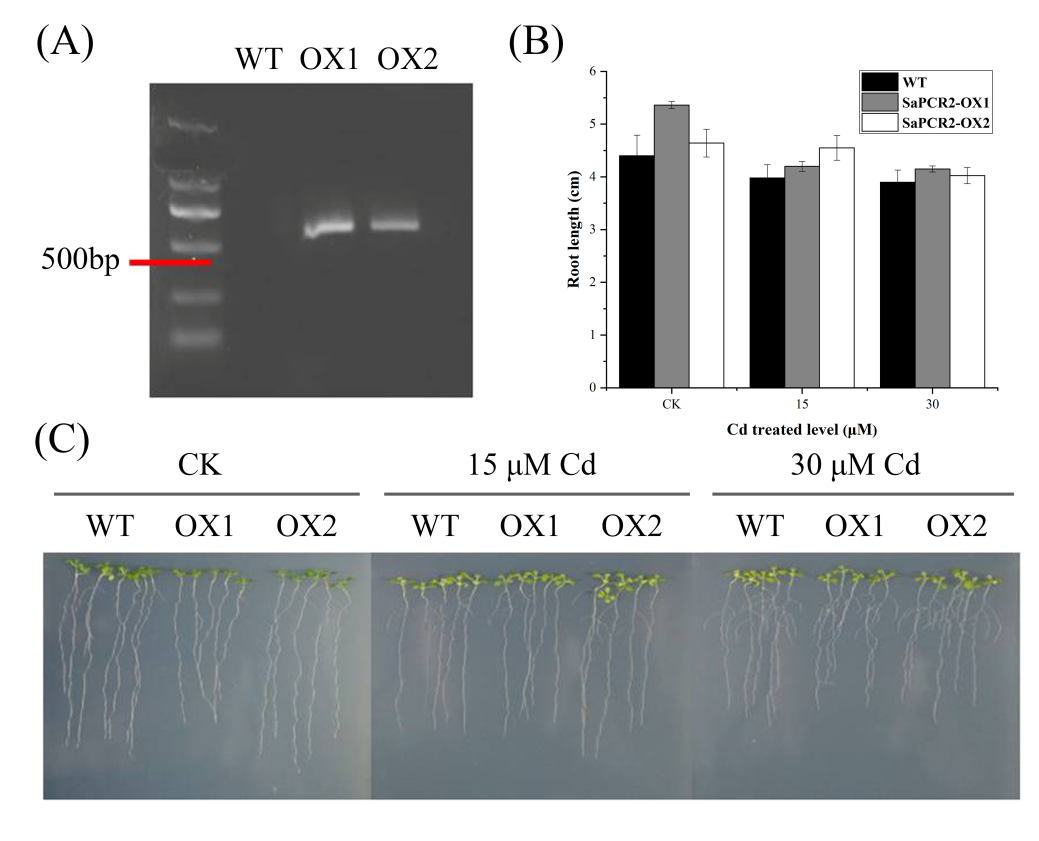


**Figure S2.** (A) Genotyping of transgenic plants. (B) Growth and (C) root length of wild type and *SaPCR2*-overexpressed transgenic *A. thaliana* lines (OX1 and OX2). Plants were cultured on 1/2 MS solid medium with 15 µM and 30 µM CdCl_2_ for 7 d, every 20 plants for one treatment, each treatment repeated three times. Error bars are ± SD of n = 3. * indicates significant difference between genotypes (*P* < 0.05).


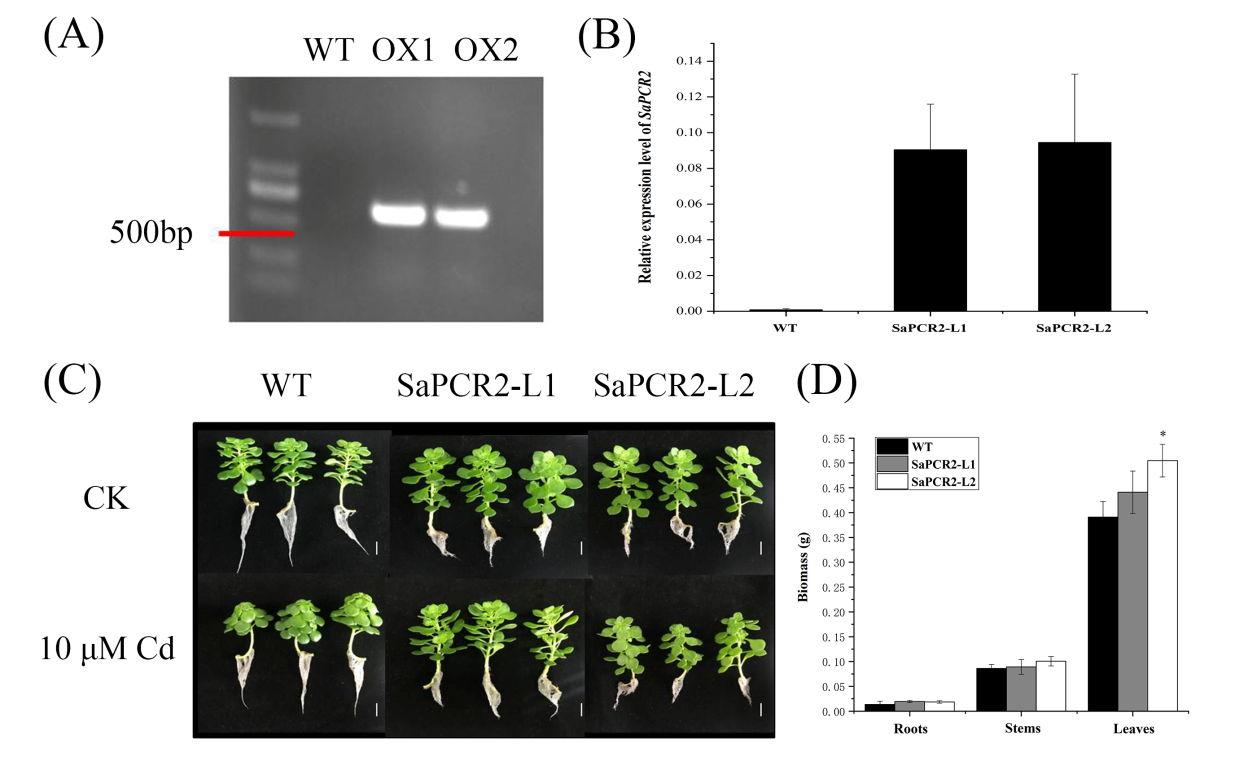


**Figure S3.** *SaPCR2* overexpressing NHE *Sedum alfredii*. (A) Genotyping of transgenic plants. (B) Relative expression level of *SaPCR2* in transgenic NHE *S. alfredii*. (C) Growth and (D) biomass of wild type and *SaPCR2*-overexpressed transgenic NHE *S. alfredii* lines (L1 and L2). Scale bar = 5 cm.
